# Palmitic Acid-Induced miR-429-3p Impairs Myoblast Differentiation by Downregulating CFL2

**DOI:** 10.3390/ijms222010972

**Published:** 2021-10-11

**Authors:** Mai Thi Nguyen, Kyung-Ho Min, Wan Lee

**Affiliations:** 1 Department of Biochemistry, Dongguk University College of Medicine, 123 Dongdae-ro, Gyeongju 38066, Korea; nguyenmainhp@gmail.com (M.T.N.); dbdlaeo112@naver.com (K.-H.M.); 2 Channelopathy Research Center, Dongguk University College of Medicine, 32 Dongguk-ro, Ilsan Dong-gu, Goyang 10326, Korea

**Keywords:** miR-429-3p, CFL2, myogenesis, differentiation, proliferation

## Abstract

MicroRNAs are known to play a critical role in skeletal myogenesis and maintenance, and cofilin-2 (CFL2) is necessary for actin cytoskeleton dynamics and myogenic differentiation. Nonetheless, target molecules and the modes of action of miRNAs, especially those responsible for the inhibitory mechanism on the myogenesis by saturated fatty acids (SFA) or obesity, still remain unclear. Here, we reported the role played by miR-429-3p on CFL2 expression, actin filament dynamics, myoblast proliferation, and myogenic differentiation in C2C12 cells. Palmitic acid (PA), the most abundant SFA in diet, inhibited the myogenic differentiation of myoblasts, accompanied by CFL2 reduction and miR-429-3p induction. Interestingly, miR-429-3p suppressed the expression of CFL2 by targeting the 3′UTR of *CFL2* mRNA directly. Transfection of miR-429-3p mimic in myoblasts increased F-actin formation and augmented nuclear YAP level, thereby promoting cell cycle progression and myoblast proliferation. Moreover, miR-429-3p mimic drastically suppressed the expressions of myogenic factors, such as MyoD, MyoG, and MyHC, and impaired myogenic differentiation of C2C12 cells. Therefore, this study unveiled the crucial role of miR-429-3p in myogenic differentiation through the suppression of CFL2 and provided implications of SFA-induced miRNA in the regulation of actin dynamics and skeletal myogenesis.

## 1. Introduction

The maintenance of skeletal muscle mass and quality by myogenesis is essential for proper physical and metabolic functioning and systemic homeostasis [1]. Skeletal myogenesis from progenitor cells to differentiated myofibers is a dynamic and well-coordinated process that underlies muscle development, growth, and regeneration [1,2]. Therefore, dysregulation of skeletal myogenesis causes multifaceted detrimental effects on health and increases morbidity and mortality rates, especially among the elderly [2]. In particular, muscle atrophy or wasting is closely associated with conditions associated with impaired myogenesis, such as senescence, oxidative stress, mitochondrial dysfunction, and enhanced ER stress [2,3]. Numerous researches have suggested that excess intake of saturated fatty acids (SFA) increases intramuscular fat infiltration and provokes lipotoxicity, such as oxidative stress, mitochondrial dysfunction, and ER stress, and thus, eventually leads to muscle wasting [4,5]. Moreover, it has been shown that dysregulation of microRNAs (miRNAs) by SFA or obesity is causally linked to muscle wasting [6,7].

MiRNAs are a class of endogenously expressed small non-coding RNAs, about 19 to 25 nucleotides long, that function as negative regulators of gene expression [8]. Although the targets and roles of miRNAs are not sufficiently understood, they are known to play significant roles in a broad range of cellular processes [8]. Accordingly, the dysregulation of miRNA is closely associated with many diseases, including cancers and metabolic diseases [9]. In the last decade, accumulating evidence has expanded our understanding of the critical roles of miRNAs in myogenesis and the pathogeneses of myopathies [10,11]. Although many miRNAs are dysregulated in obesity and are implicated in muscle wasting [6,7,12], the mechanisms by which the inhibition of myogenic differentiation through SFA-induced miRNAs occurs are poorly understood.

Actin is the most abundant intracellular protein and participates in numerous cellular activities via the dynamic remodeling of actin filaments [13,14]. Actin-binding and regulatory proteins orchestrate the cytoskeletal dynamics essential for myoblast proliferation, differentiation, and myofiber formation [13,14]. Notably, cofilin 2 (CFL2) is a skeletal muscle-specific actin-binding protein and member of the actin-depolymerizing factor (ADF)/cofilin family, which disassembles filamentous actin (F-actin) [15]. Growing evidence suggests that CFL2 is a critical player in muscle development and maintenance by regulating actin cytoskeleton dynamics in skeletal muscle [16]. CFL2 knockout in mice exhibited skeletal muscle weakness and aberrant sarcomere architecture along with F-actin accumulations due to a loss of actin depolymerization activity [17]. In another study, CFL2 knockout resulted in progressive muscle degeneration characterized by fiber size disproportion, sarcoplasmic protein aggregates, and mitochondrial abnormalities [18]. Recently, we showed that CFL2 knockdown in myoblasts impeded myogenic differentiation by promoting cell proliferation and suppressing myogenic regulatory factors [19]. Although previous studies have suggested that CFL2 is required for skeletal myogenesis and function, nothing is known of CFL2 regulation by miRNA during myogenic differentiation.

This study investigated the effects of miR-429-3p on CFL2 expression and myogenic differentiation in C2C12 myoblasts. Palmitic acid (PA), the most abundant SFA in diet, inhibited myogenic differentiation, downregulated CFL2 expression, and induced miR-429-3p. Interestingly, miR-429-3p was found to target the 3′UTR of *CFL2* and suppressed the protein expression of CFL2. To determine the significance of miR-141-3p in myogenesis, we investigated the effect of miR-429-3p on myoblast proliferation, myogenic factor expression, and myogenic differentiation using functional and cytochemical analysis. Our study highlights the importance of miR-429-3p targeting CFL2 during myoblast differentiation and suggests a putative miRNA-mediated regulatory mechanism for myogenesis in the background of obesity.

## 2. Results

### 2.1. PA Inhibited Myoblast Differentiation and Induced the Expression of miR-429-3p

**Figure 1:**
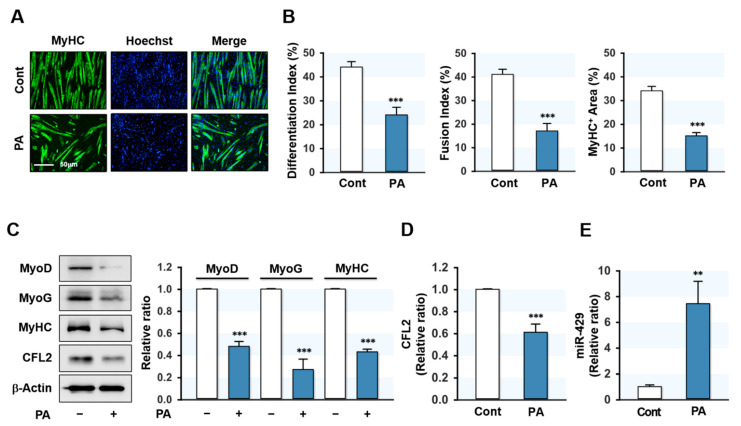
PA inhibited myoblast differentiation and induced miR-429-3p expression. To analyze the effect of PA on myogenic differentiation, C2C12 cells were allowed to differentiate for five days after treatment with PA (100 μM, 24 h). (**A**) Immunocytochemistry staining with a MyHC (green) antibody. Hoechst (blue) was used to stain nuclei. Scale bar: 50 μm. (**B**) The analysis of differentiation index, fusion index, and MyHC-positive area. (**C**,**D**) Immunoblot analysis of myogenic factors (MyoD, MyoG, and MyHC) and CFL2. β-Actin was used as a loading control. (**E**) *q*RT-PCR analysis of miR-429-3p expression. *q*RT-PCR and immunoblot results are shown as ratios vs. normal controls. All results are presented as means ± SEM (*n* > 3), and levels of significance are presented as ** *p* < 0.01; *** *p* < 0.001 vs. controls.

To determine the effect of SFA on myoblast differentiation, C2C12 cells were treated with PA (100 μM) for 24 h prior to the induction of differentiation. Myoblast differentiation was then investigated by analyzing myotube formation and myogenic factor expressions up to differentiation day 5. Immunocytochemical analysis showed that PA markedly reduced the MyHC-positive area and inhibited myoblast differentiation and fusion in C2C12 cells (Figure 1A,B). This result is consistent with our previous findings [20] and implies that inhibition of myogenic differentiation by PA was established in this study. Subsequently, the expressions of myogenic factors were analyzed in control and PA-treated myoblasts. As shown in Figure 1C, the expressions of MyoD, MyoG, and MyHC were markedly reduced by PA. These results indicated that PA suppressed myogenic factor expression and impaired the myogenic differentiation of progenitor cells. Since we previously found that CFL2 is required for myogenesis [19], we investigated whether PA inhibits CFL2 expression in C2C12 myoblasts. As shown in Figure 1C,D, PA dramatically reduced the expression of CFL2. To determine how PA suppressed CFL2 expression and myogenic differentiation, we hypothesized that specific miRNAs induced by PA might contribute to the downregulation of CFL2. According to the microarray analysis of miRNA expression in myoblasts, many miRNAs were induced by PA (data not shown). Among those, miR-429-3p was chosen for further experiments because CFL2 was suggested as a potential target of miR-429-3p by miRNA target prediction analysis, and *q*RT-PCR analysis confirmed the drastic upregulation of miR-429-3p expression by PA (Figure 1E). Collectively, PA was found to impair the differentiation of myoblasts, suppress CFL2 expression, and induce the expression of miR-429-3p.

### 2.2. MiR-429-3p Directly Targeted the 3′UTR of CFL2

**Figure 2:**
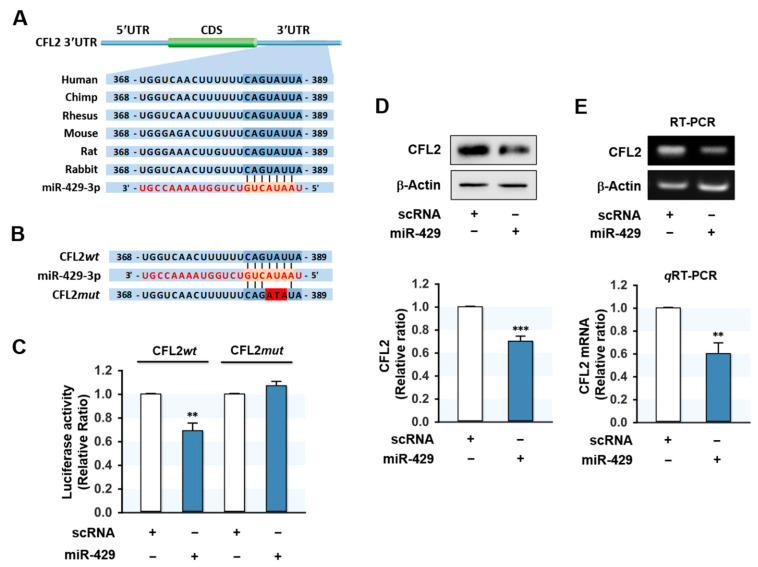
CFL2 is a target gene of miR-429-3p. (**A**) Diagram showing that the 3′UTR fragment of *CFL2* contains a miR-429-3p binding site. (**B**) Sequences of the wild type (CFL2*wt*) and mutant (CFL2*mut*) *CFL2* 3′UTR for miR-429-3p binding. (**C**) pmirGLO-CFL2*wt* or pmirGLO-CFL2*mut* plasmids were co-transfected with scRNA or miR-429-3p into C2C12 cells. Luciferase reporter assays were performed 24 h later. (**D**) Immunoblot analysis of CFL2 at 24 h after transfection with 200 nM of scRNA or miR-429-3p mimic. (**E**) RT-PCR (upper) and *q*RT-PCR (lower) analysis of CFL2 expression at 24 h after transfection. The level of expression was normalized to the amount of ß-Actin. *q*RT-PCR and immunoblot results are shown as relative ratios vs. scRNA. All results are presented as means ± SEM (*n* > 3), and levels of significance are presented as ** *p* < 0.01; *** *p* < 0.001 vs. scRNA.

Since CFL2 protein expression was inversely related to miR-429-3p level in myoblasts, we investigated whether CFL2 is a direct target of miR-429-3p. In silico analysis using TargetScan and PicTar indicated that the 3′UTR of *CFL2* includes a tentative binding site with a high affinity for the seed sequence of miR-429-3p (Figure 2A). To confirm direct targeting of miR-429-3p on *CFL2*, we produced pmirGLO luciferase reporter constructs containing a 3′UTR segment of *CFL2* with either the predicted binding site of miR-429-3p (wild type; CFL2*wt*) or a mutated binding site (CFL2*mut*) (Figure 2B). Subsequently, a pmirGLO construct and either miR-429-3p mimic or scRNA were co-transfected in C2C12 myoblasts. As we expected, miR-429-3p mimic reduced luciferase activity of the wild type (CFL2*wt*), whereas a mutant construct in the miR-429-3p binding site (CFL2*mut*) almost completely abolished the effect of miR-429-3p on the luciferase activity of CFL2*wt*. Thus, binding between miR-429-3p mimic and the 3′UTR of *CFL2* was confirmed by luciferase reporter analysis. Next, to examine whether miR-429-3p induction inhibits CFL2 expression in myoblasts, we transfected C2C12 myoblasts with scRNA or miR-429-3p mimic and then analyzed CFL2 protein and mRNA expressions. As shown in Figure 2D, transfection with miR-429-3p mimic decreased CFL2 protein level significantly as compared with scRNA transfection. In addition, *CFL2* mRNA expression was also reduced by miR-429-3p mimic as determined by RT-PCR and *q*RT-PCR (Figure 2E), indicating that miR-429-3p is a negative regulator of CFL2 expression.

### 2.3. MiR-429-3p Augmented F-Actin Formation and Nuclear Yes-Associated Protein (YAP)

**Figure 3:**
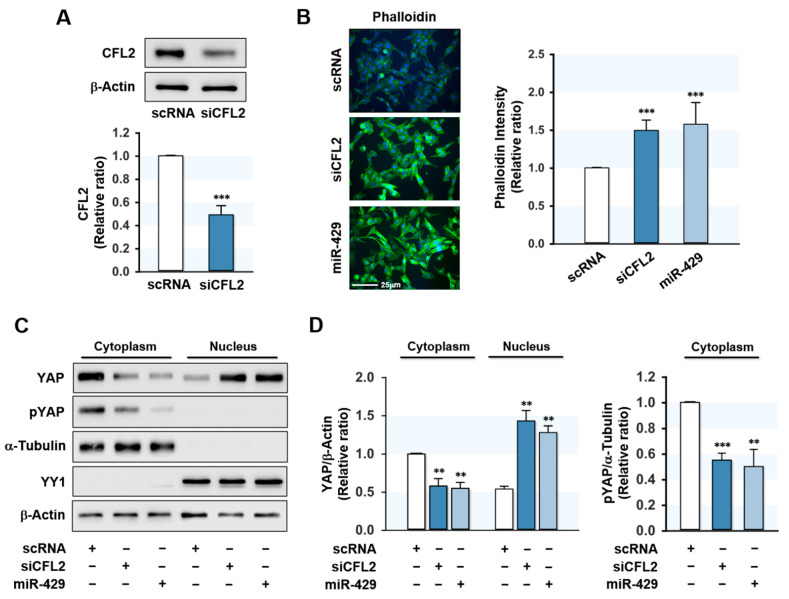
MiR-429-3p mimic elevated F-actin and nuclear YAP level. C2C12 myoblasts were transfected with 200 nM of scRNA, siCFL2, or miR-429-3p mimic. (**A**) After 24 h transfection, CFL2 protein expressions were determined by immunoblotting. (**B**) Representative images of FITC-phalloidin (green) representing F-actin staining. Scale bar: 25 μm. Phalloidin intensity was analyzed by the ImageJ program. (**C**,**D**) Cytoplasmic and nuclear fractions of myoblasts were separated after 24 h of transfection, and subcellular fractionation was confirmed using cytoplasmic (α-Tubulin) or nuclear (YY1) markers. The distribution of YAP and phospho-YAP (pYAP) was determined by immunoblotting and densitometry. Immunoblot results are shown as relative ratios vs. scRNA controls. All results are presented as means ± SEM (*n* > 3), and levels of significance as ** *p* < 0.01; *** *p* < 0.001 vs. scRNA.

CFL2 modulates actin cytoskeleton dynamics by depolymerizing actin filaments [21] and CFL2 downregulation increases F-actin formation in myoblasts [19]. Therefore, we next investigated the effect of miR-429-3p on F-actin formation in myoblasts. Transfection with CFL2 siRNA (siCFL2) suppressed CFL2 protein level by ~50% as compared with scRNA control (Figure 3A), and transfection with miR-429-3p mimic efficiently elevated (>300-fold) the cellular level of miR-429-3p in myoblasts (data not shown). Under our experimental conditions, the cytochemical analysis showed that both siCFL2 and miR-429-3p mimic dramatically increased the accumulation of F-actin (Figure 3B). Given that total actin levels remained constant in all groups throughout differentiation, these increases in F-actin seemed to result from a defect in actin depolymerization. Therefore, it is suggested that miR-429-3p strongly promotes the formation of F-actin stress fibers by reducing CFL2 levels in myoblasts. F-actin is known to reduce phosphorylation of the transcriptional coactivator YAP in the cytoplasm and induce the nuclear translocation of YAP, which activates proliferative transcriptional programs in the Hippo signaling pathway [22,23]. To analyze whether miR-429-3p increases nuclear translocation of YAP, we evaluated the phosphorylation (Y357) and nuclear level of YAP in myoblasts. Transfection with miR-429-3p mimic drastically decreased the phosphorylation of YAP in the cytoplasm and redistributed YAP to the nucleus from the cytosol (Figure 3C,D), implying that miR-429-3p activates YAP in myoblasts.

### 2.4. MiR-429-3p Promoted Myoblast Proliferation and Cell Cycle Progression

**Figure 4:**
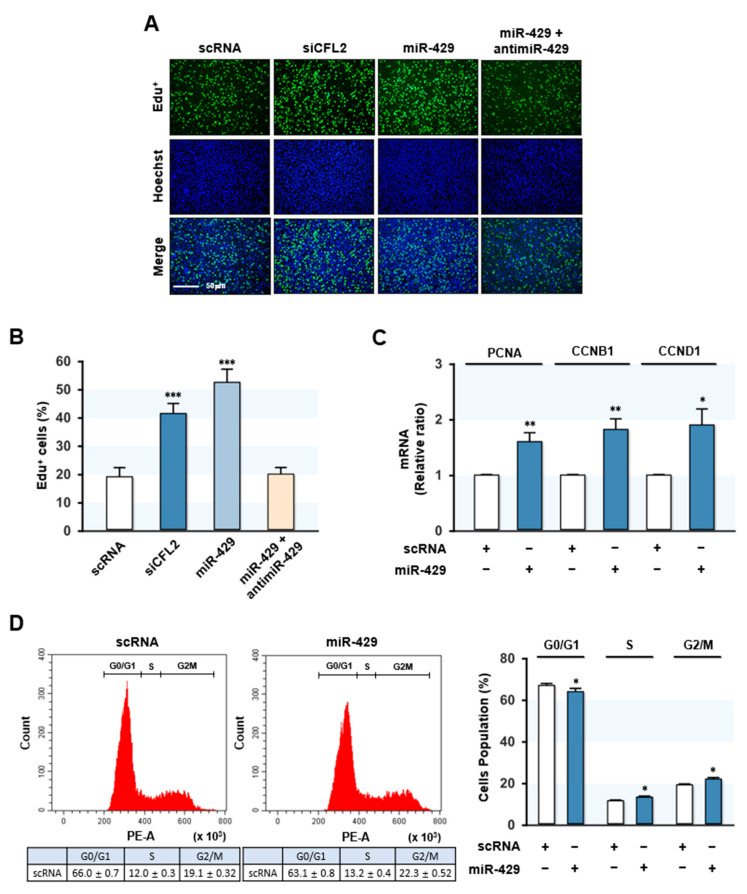
MiR-429-3p mimic promoted myoblast proliferation and cell cycle progression. C2C12 myoblasts were transfected with scRNA control, siCFL2, miR-429-3p mimic, or antimiR-429 (200 nM). (**A**) EdU assays were performed after 24 h of transfection. Cells were labeled with 10 µM of EdU (green), and nuclei were stained with Hoechst (blue). Scale bar: 50 μm. (**B**) Percentages of EdU-positive cells were analyzed by the ImageJ program. (**C**) *q*RT-PCR analysis of the expression levels of PCNA, CCNB1, and CCND1. Expression levels were normalized versus U6. (**D**) Flow cytometry with scatter plot of cell cycle analysis after transfection with scRNA or miR-429-3p mimic. All results are presented as means ± SEM (*n* > 3), and levels of significance as * *p* < 0.05; ** *p* < 0.01; *** *p* < 0.001 vs. scRNA.

Previously, it was demonstrated that knockdown of CFL2 inhibited myoblast differentiation by activating myoblast proliferation and cell cycle progression [19]. Since miR-429-3p reduced CFL2 expression and increased nuclear YAP in myoblasts, we investigated the role of miR-429-3p in myoblast proliferation and the cell cycle. Proliferation analysis based on EdU incorporation revealed that siCFL2 substantially increased EdU-positive myoblast numbers as compared with scRNA (Figure 4A,B), indicating that CFL2 suppression stimulated myoblast proliferation. As was expected, miR-429-3p mimic transfection significantly enhanced EdU incorporation into myoblasts, whereas co-transfection with antimiR-429-3p nearly rescued the increased EdU incorporation observed in miR-429-3p-mimic-treated cells (Figure 4A,B). These results demonstrated that miR-429-3p increased myoblast proliferation. Next, the mRNA expressions of proliferating cell nuclear antigen (PCNA) and cyclins (CCNB1 and CCND1), which are known to induce cell proliferation and cell cycle progression [24,25,26], were analyzed. *q*RT-PCR analysis showed that PCNA expression was significantly upregulated in myoblasts transfected with miR-429-3p mimic (Figure 4C). Moreover, this transfection also dramatically increased the expressions of CCNB1 and CCND1 (Figure 4C). We also assessed the effect of miR-429-3p on the cell cycle by fluorescence-activated cell sorting. MiR-429-3p mimic transfection lowered the proportion of cells in the G0/G1 phase but increased it in the S and G2/M phase (Figure 4D). Collectively, miR-429-3p mimic promoted myoblast proliferation and cell cycle progression in myoblasts.

### 2.5. MiR-429-3p Decreased the Expressions of Myogenic Factors

**Figure 5:**
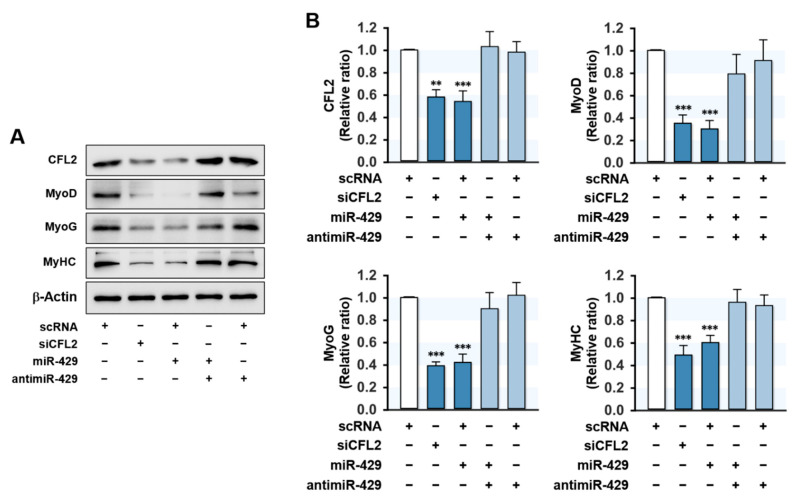
MiR-429-3p mimic suppressed myogenic factor expression. Cells were transfected with 200 nM of scRNA, siCFL2, miR-429-3p mimic, or antimiR-429-3p and were allowed to differentiate for three days. (**A**) Representative immunoblots showing the expressions of CFL2, MyoD, MyoG, and MyHC. (**B**) Quantitative analysis of CFL2 and myogenic factors (MyoD, MyoG, and MyHC) protein levels. Immunoblot results are shown as relative ratios versus scRNA controls. All results are presented as means ± SEM (*n* > 3), and levels of significance as ** *p* < 0.01; *** *p* < 0.001 vs. scRNA.

Since proliferation arrest and cell cycle exit are required conditions for myogenic differentiation [27], the promotion of proliferation by miR-429-3p in myoblasts may inextricably lead to the suppression of myogenic differentiation. Hence, we investigated whether the induction of miR-429-3p suppresses the expressions of myogenic factors. C2C12 myoblasts were transfected with scRNA, siCFL2, miR-429-3p mimic, or antimiR-429-3p, and the expressions of MyoD, MyoG, and MyHC were determined on differentiation day 3. As shown in Figure 5A,B, siCFL2 transfection reduced CFL2 level by about 50% versus scRNA control and markedly decreased the protein levels of myogenic factors (MyoD, MyoG, and MyHC). Interestingly, transfection with miR-429-3p mimic also suppressed CFL2 level markedly and reduced the expressions of MyoD, MyoG, and MyHC versus scRNA controls (Figure 5A,B). In addition, co-transfection with antimiR-429-3p and miR-429-3p mimic nearly rescued the inhibitory effect of miR-429-3p on myogenic factor expression (Figure 5A,B). This inhibitory effect of miR-429-3p on myogenic factors was ascribed to the direct suppression of CFL2 in C2C12 myoblasts because the 3′UTRs of myogenic factors do not possess a putative binding site for miR-429-3p. Thus, miR-429-3p suppresses myogenic factor expression in C2C12 myoblasts.

### 2.6. MiR-429-3p Impaired Myogenic Differentiation

**Figure 6:**
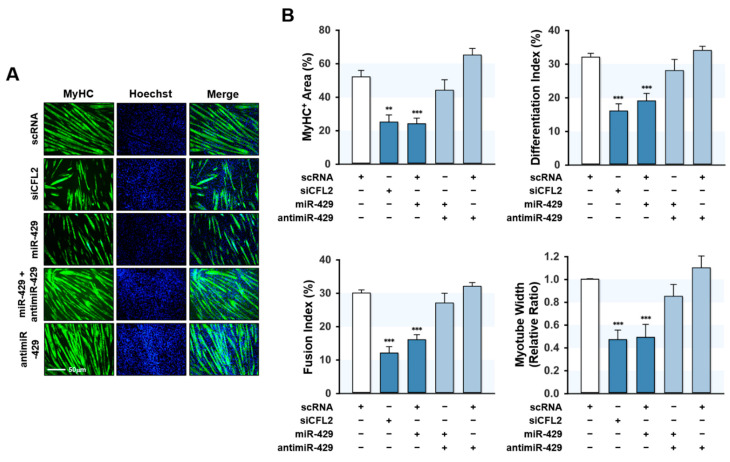
MiR-429-3p mimic impaired myogenic differentiation and myotube formation. After transfection with scRNA control, siCFL2, miR-429-3p mimic, or antimiR-429-3p (200 μM) for 24 h, C2C12 myoblasts were differentiated for five days. (**A**) MyHC (green)-positive myotubes were obtained by immunofluorescence staining, and nuclei were counterstained with Hoechst (blue). Scale bar: 50 μm. (**B**), MyHC-positive area, differentiation index, fusion index, and myotube width were determined as described in the Methods Section. All results presented are means ± SEM (*n* > 3), and levels of significance are indicated by ** *p* < 0.01; *** *p* < 0.001 vs. scRNA.

Since miR-429-3p was found to increase myoblast proliferation and suppress the expression of myogenic factors, we next investigated whether miR-429-3p inhibits myoblast differentiation. Myoblasts were transfected with scRNA, siCFL2, miR-429-3p mimic, or antimiR-429-3p and cultured in a differentiation medium for five days (Figure 6). Myoblast differentiation was analyzed by immunocytochemistry using MyHC antibody and subjected to further quantitative image analysis. Knockdown of CFL2 with siCFL2 significantly reduced myotube formation in myoblasts as determined by MyHC immunofluorescence (Figure 6A). Furthermore, differentiation indices, fusion indices, percentage areas of MyHC-positive cells, and myotube widths suggested that CFL2 knockdown was causally linked to impaired myoblast differentiation (Figure 6B). Similarly, transfection with miR-429-3p mimic also inhibited myoblast differentiation as assessed by immunocytochemistry and image analysis (Figure 6A,B). Additionally, co-transfection with antimiR-429-3p almost completely eliminated the inhibitory effect of miR-429-3p mimic on myoblast differentiation and myotube formation (Figure 6A,B). These results suggest that miR-429-3p negatively regulates myogenic differentiation and myotube formation in C2C12 myoblasts.

## 3. Discussion

Although increasing evidence indicates that miRNAs are essential regulators of myogenesis [10,11], the mechanisms whereby miRNAs induced by SFA or obesity are linked to the suppression of myogenic differentiation remain poorly understood. Here, for the first time, we demonstrated the critical roles played by miR-429-3p on CFL2 expression, myoblast proliferation, and myogenic differentiation. The following are the key contributions this study makes to current understanding: (i) PA inhibited myoblast differentiation accompanied with CFL2 reduction and miR-429-3p induction. (ii) Transfection with miR-429-3p mimic suppressed CFL2 expression by directly targeting the 3′UTR of *CFL2*. (iii) MiR-429-3p mimic increased F-actin and nuclear YAP in myoblasts, thereby promoting cell cycle progression and myoblast proliferation. (iv) MiR-429-3p mimic markedly suppressed myogenic factors and impaired myoblast differentiation. Therefore, this study reveals that miR-429-3p is a critical regulator of myogenic differentiation and provides the miRNA-mediated myogenic regulatory mechanism in association with SFA and obesity.

MiR-429-3p is a member of the miR-200 family and consists of five highly conserved miRNAs on two distinct genomic regions: the miR-200b/200a/429 cluster and the miR-200c/141 cluster [28]. Members of the miR-200 family have been shown to have pleiotropic regulatory functions in tissue homeostasis and oncogenesis [28,29]. Several studies have demonstrated the involvement of miR-429-3p in a wide range of oncogenic properties, including growth advantage, invasiveness, and metastasis [28,29,30,31,32,33]. In addition, this study showed the regulatory roles of miR-429-3p on actin dynamics, myoblast proliferation, and myogenic differentiation. Myoblast proliferation and myogenic differentiation have been well established as being inversely related during myogenesis, which indicates proliferation arrest is a prerequisite for the myogenic differentiation in myoblasts [1]. In this regard, it is critical to note that miR-429-3p promoted cell cycle progression and proliferation with the upregulation of PCNA, CCNB1, and CCND1 in myoblasts. Recent research on the effect of miR-429-3p on the cell cycle and proliferation in various malignancies supports our results. The oncogenic role of miR-429-3p has been confirmed in diverse organs, including the liver, lung, stomach, and breast [28,29]. Moreover, miR-429-3p overexpression was reported to promote preadipocyte proliferation and inhibited adipogenic differentiation [30]. MiR-429-3p upregulation activated cell progression, proliferation, migration, and invasion in lung carcinoma cells [31], whereas miR-429-3p knockdown suppressed proliferation in lung carcinoma cells [32] and prostate cancer cells [33]. Accordingly, the effect of miR-429-3p on myoblast proliferation and the cell cycle is inextricably related to the suppression of myogenic differentiation observed in myoblasts.

Then how does miR-429-3p induce myoblast proliferation and cell cycle progression? One of the essential findings in this study is that miR-429-3p augmented F-actin by suppressing CFL2 expression (Figure 3). CFL2 plays a crucial role in actin dynamics by depolymerizing F-actin, and thus, it regulates mechanical stress in the cytoskeleton [21,34]. Actin cytoskeleton dynamics and mechanotransduction have been suggested as regulatory mechanisms for YAP activation in the Hippo signaling pathway, which modulates organ and tissue size via proliferation, differentiation, and apoptosis [35]. In the Hippo signaling pathway, the nuclear translocation of cytosolic YAP and TAZ triggers proliferative and anti-apoptotic transcriptional processes [36]. Recently, F-actin was found to reduce YAP phosphorylation and consequently increase the nuclear translocation of YAP [22]. In addition, proteins that cleave F-actin, such as CFL and Gelosin, serve as negative regulators of YAP by increasing its phosphorylation and degradation [37]. Accordingly, CFL-mediated actin remodeling is intimately linked to the induction of cell proliferation through the nuclear translocation of YAP [34,37]. In our previous study, knockdown of CFL2 by siRNA increased F-actin formation and promoted cell cycle progression and proliferation in myoblasts [19]. In another study, F-actin inducer jasplakinolide increased YAP translocation to the nucleus, whereas the actin depolymerizer cytochalasin D had the opposite effect in cardiomyocytes [38]. Therefore, our results suggest that the effect of miR-429-3p on cell proliferation is primarily due to the perturbation of actin dynamics caused by reduced CFL2 expression. Collectively, it is suggested that the induction of miR-429-3p mechanistically increases F-actin formation and nuclear YAP level by suppressing CFL2, thereby promoting cell cycle progression and cell proliferation.

The molecular regulatory mechanisms responsible for the induction of miR-429-3p by PA in myoblasts are still an open question. However, certain transcription factors activated by PA or obesity may trigger the transcriptional activation of miR-429-3p. Based on the results from the high-fat-diet (HFD)-induced obesity rodent model, miR-429-3p is known to be upregulated in the liver [39,40,41]. Interestingly, previous evidence has suggested that tumor necrosis factor (TNF), receptor-associated factor 6 (TRAF6), and enhancer of the zeste homolog 2 (EZH2) are key players in the upregulation of miR-429-3p in HFD-induced obesity [42]. TRAF6, an adaptor protein that possesses E3 ubiquitin ligase activity, is increased in HFD-induced obesity [43,44] and increases ubiquitination and degradation of EZH2 to reduce EZH2 level [42,45]. EZH2 has been documented to repress miR-429-3p expression by binding to the miR-429-3p promoter [42,46,47]. Therefore, it is suggested that enhancement of TRAF6 expression in obesity could increase degradation of EZH2, thereby promoting upregulation of miR-429-3p. In addition, TRAF6 also plays a critical role in the activation of nuclear transcription factors associated with adipogenesis and obesity, such as NF-κB and AP-1 [48,49]. In silico analysis of transcription factor binding sites showed that the promoter regions of miR-429-3p contain tentative binding sites for NF-κB and AP-1. These transcription factors play critical roles in the regulation of inflammation and adipogenesis, and their activations are linked to a high-fat diet and obesity [50,51]. Since the expression of TRAF6 and miR-429-3p was drastically increased in HFD-fed mice and the oleic acid/palmitic acid (OA/PA)-treated cells, it appears that the activations of TRAF6, NF-κB, and AP-1 may contribute to the upregulation of miR-429-3p by PA. Further research is needed to determine how transcriptional factors regulate miR-429-3p. Nonetheless, results available to date indicate that miR-429-3p may be a novel mediator in the association between obesity and muscle mass reduction.

## 4. Materials and Methods

### 4.1. Cell Culture and PA Treatment

C2C12 myoblasts, a murine-derived muscle progenitor cell line from ATCC (Manassas, VA, USA), were maintained in a growth medium (Dulbecco’s modified Eagle’s medium (DMEM) containing 10% fetal bovine serum and 1% penicillin/streptomycin) (Gibco, Carlsbad, CA, USA) at 37 °C in a humidified incubator with 5% CO_2_. One day before transfection, cells were seeded in a 6-well plate at 1.3 × 10^5^ cells/well to 80–90% confluence. Cells were transiently transfected using Lipofectamine 2000 (Invitrogen, Waltham, MA, USA) according to the manufacturer’s instructions. One day later, the growth medium was changed to a differentiation medium (DMEM containing 2% horse serum and 1% penicillin/streptomycin) to induce myogenic differentiation. When necessary, cells were treated with BSA-conjugated PA (100 μM) for 24 h in growth medium before differentiation as described previously [20]. Unless otherwise stated, all reagents and materials were purchased from Sigma Aldrich, St. Louis, MO, USA.

### 4.2. Transfection of miRNA Mimic

Scrambled control RNA (scRNA), CFL2 siRNA (siCFL2), miR-429-3p mimic, or antimiR-429-3p (an inhibitor of miR-429-3p; a 2′-O-methyl-modified antisense oligonucleotide against mature miR-429-3p) (Genolution, Seoul, Korea) were transfected into C2C12 myoblasts using Lipofectamine 2000 (Invitrogen, Waltham, MA, USA) at a concentration of 200 nM. Oligonucleotide sequences are listed in Table S1.

### 4.3. RNA Preparation and Quantitative Real-Time PCR (qRT-PCR)

Total RNAs of C2C12 cells were obtained using Qiazol reagent (Qiagen, Düsseldorf, Germany) 24 h after transfection and were purified with a miRNeasy Mini Kit (Qiagen). RNA concentrations were determined using a UV-1700 PharmaSpec spectrophotometer (Shimadzu, Japan). cDNAs were synthesized using a miScript II RT Kit (Qiagen). To determine mRNA and miRNA expressions, SYBR Green I and iTaq polymerase (Promega, Madison) were used for *q*RT-PCR in a LightCycler 480 (Roche Applied Science, Penzberg, Germany). RT-PCR and *q*RT-PCR primers and reaction conditions are listed in Table S2. Relative gene expressions were determined using the 2^−ΔΔCt^ method and normalized versus GAPDH or U6.

### 4.4. Dual-Luciferase Reporter Assay

Murine *CFL2* 3′UTR segment (238 nt) containing a binding site for miR-429-3p was chemically synthesized by RT-PCR and subcloned into pmirGLO (Promega) to produce *CFL2* 3′UTR-wild-type (CFL2*wt*) or *CFL2* 3′UTR-mutant (CFL2*mut*) plasmids using specific primers as described in Table S2. C2C12 cells were cultured in 12-well plates for one day, and then CFL2*wt* or CFL2*mut* plasmid was co-transfected with scRNA or miR-429-3p mimic into cells using Lipofectamine 2000 (Invitrogen). Dual-luciferase reporter gene assays were performed 24 h after transfection, as described previously [52].

### 4.5. Immunoblot Analysis

For protein preparation, C2C12 cells were lysed and solubilized with PBS containing 2% Triton-X 100 and 0.1% phosphatase inhibitor cocktail, as previously described [53]. For nuclear/cytoplasmic protein fractionation, the NE-PER Nuclear and Cytoplasmic Extraction Reagents (Thermo Fisher Scientific, Waltham, MA, USA) were used by following the manufacturer’s protocol. Proteins (20 μg) were resolved by SDS-PAGE and subjected to immunoblot analysis using specific antibodies (Table S3). Blots were visualized using Femto reagent (Thermo Fisher Scientific), detected by Fusion Solo (Vilber, France), and the intensity of immunoblots was analyzed using Evolution Capt software (Vilber, France).

### 4.6. Immunofluorescence Analysis

C2C12 cells were allowed to differentiate for five days after scRNA, siCFL2, miR-429-3p mimic, or antimiR-429-3p transfection fixed, permeabilized, and blocked them, as described in the previous study [19]. The fixed cells were incubated with MyHC antibodies (1:100 dilution) overnight at 4 °C and then treated with Alexa-488-conjugated goat anti-mouse antibody (Invitrogen) for another 1.5 h. Hoechst 33342 (Invitrogen) was added for 15 min to stain nuclear DNA. To detect the F-actin cytoskeleton, cells were fixed, permeabilized, and stained with FITC-coupled phalloidin (P5282, Sigma, New York, NY, USA) after 24 h of transfection, as described previously [19]. Images were taken and analyzed by a fluorescence microscope (Leica, Germany). Differentiation indices were calculated by expressing numbers of nuclei in MyHC-positive myotubes as percentages of total nuclei, and fusion indices by expressing numbers of nuclei in myotubes (three or more nuclei) as percentages of total nuclei. Numbers of myotubes, myotube widths, and MyHC-positive areas were measured using ImageJ 1.53j. Experiments were carried out at least three times, and five randomly selected fields were examined per experiment.

### 4.7. Cell Proliferation Assays

EdU assays were performed to evaluate cell proliferation using the Click-iT™ EdU Cell Proliferation Kit (Invitrogen). Briefly, after 24 h transfection, EdU was added to the medium at 10 μM and incubated for 4 h. Cells were then fixed with formaldehyde (4%) for 10 min, permeabilized with Triton X-100 (0.3%) in PBS for 15 min, incubated with 0.3 mL of Click-iT reaction cocktail for 20 min, and nuclei were labeled with Hoechst 33342 for an additional 15 min. All images were obtained using a fluorescent microscope (Leica, Germany). Percentages of EdU-positive cells and total numbers of nuclei were analyzed by Image J Software. All experiments were conducted at least three times using at least five randomly selected fields per experiment.

### 4.8. Flow Cytometry

After transfection for 24 h, C2C12 cells were detached using trypsin EDTA into 2 mL tubes, retrieved by centrifugation at 3000 rpm for 5 min, washed with PBS three times, and fixed in 70% ethanol overnight at 4 °C. The fixed cells were then incubated in a cycle kit solution (C03551, Beckman Coulter, Brea, CA, USA) for 20 min at room temperature in the dark. Cell cycles were analyzed using a CytoFLEX (Beckman Coulter, Brea, CA, USA).

### 4.9. Database and Statistical Analysis

Putative binding sites for miR-429-3p on the 3′UTR of *CFL2* mRNA were identified using publicly available bioinformatics software (TargetScan: www.targetscan.org, Pictar: pictar.mdc-berlin.de). Results are presented as the means ± standard errors of at least three independent experiments. The significances of differences were determined using the Student’s *t*-test for unpaired data.

## 5. Conclusions

This study shows that miR-429-3p plays an essential role in CFL2 expression and myogenic differentiation in C2C12 cells. PA inhibited differentiation of myoblasts and provoked CFL2 reduction and miR-429-3p induction. Interestingly, miR-429-3p suppressed CFL2 expression by targeting the 3′UTR of *CFL2* mRNA. Transfection of miR-429-3p mimic transfection augmented F-actin and increased nuclear YAP levels, and thus, promoted myoblast proliferation and impaired myoblast differentiation. This inhibitory effect of miR-429-3p on myogenic differentiation was ascribed to the direct inhibition of CFL2 expression. These effects of miR-429-3p on CFL2 expression and myogenic differentiation suggest a novel miRNA-mediated mechanism that regulates myogenesis in the background of obesity. Moreover, PA-mediated dysregulation of myomiRs has recently been postulated as a mechanism of sarcopenic obesity and a therapeutic target [54]. Thus, miR429-3p and other miRNAs regulated by PA may be vital mediators between obesity and muscle wasting and may provide a means of developing practical diagnostic and therapeutic approaches for muscle wasting and sarcopenic obesity.

## Data Availability

The data presented in this study are available on request from the corresponding author.

## Supplementary Materials

The following are available online at https://www.mdpi.com/article/10.3390/ijms222010972/s1.
