# Supplementary material for: Palmitic Acid-Induced miR-429-3p Impairs Myoblast Differentiation by Downregulating CFL2
Source: Int J Mol Sci. 2021 Oct 11;22(20):10972. doi: 10.3390/ijms222010972 (PMC8535884; doi:10.3390/ijms222010972)
Supplement: Supplementary file 1 [file ijms-22-10972-s001.zip › Table S1-3.pdf]

Table S1. Oligonucleotide sequences for transfection

Gene	Primer sequence (5′-3′)
scRNA (control RNA)	UGGAAGACUAGUGAUUUUGUUGUU
siCFL2	GCUCUAAAGAUGCCAUUAAUU
miR-429-3p	UAAUACUGUCUGGUAAAACCGU
antimiR-429	Genolution

Table S2. Primer lists and PCR conditions for *q* RT-PCR, RT-PCR and cloning

(A) Mouse primer lists for *q* RT-PCR and RT-PCR

Gene	Primer sequence (5′-3′)		Product size	Annealing Temperature	Concentration		Cycle
					cDNA	Primer	
miR-429	F.P	TAATACTGTCTGGTAAAACCGT	90	55	2 ng/μl	0.5 μM	40
miRNA universal Primer	R.P	miScript universal primer (Qiagen)					
U6	F.P	CTCGCTTCGGCAGCACA	94				
	R.P	AACGCTTCACGAATTTGCGT					
β-Actin	F.P	TCACCCACACTGTGCCCATCTACGA	348	58			
	R.P	GGATGCCACAGGATTCCATACCCA					
CFL2	F.P	CCGACCCCTCCTTCTTCTCG	100	58			
	R.P	GTAACTCCAGATGCCATAGTG					
Ccnd1	F.P	ACCAATCTCCTCAACGACCG	228	58			
	R.P	ACGGAAGGGAAGAGAAGGG					
Ccnb1	F.P	GAGCTATCCTCATTGACTGG	125	58			
	R.P	CATCTTCTTGGGCACACAAC					
PCNA	F.P	GAACCTGCAGAGCATGGACTC	201	58			
	R.P	GGTGTCTGCATTATCTTCAGCCC					

(B) Primer lists for wild-type and mutant 3'UTR cloning

Gene	Primer sequence (5′-3′)		Product size	Annealing Temperature	Concentration		Cycle
					cDNA	Primer	
Hsa-CFL2-3′UTR wild type	F.P	ATTATCCACTGGTCAGATGGTC	238	58	2 ng/μl	0.5 μM	35
	R.P	AGGTACTAGGATAAGTTGATGACACAG					
Hsa-CFL2-3′UTR mutation	F.P	ATTATCCACTGGTCAGATGGTC	56				
	R.P	CAAGTGCCAACTATAAATATATCTGAAA					
	F.P	TTTCAGATATATTTATAGTTGGCACTTG	210				
	R.P	AGGTACTAGGATAAGTTGATGACACAG					

Table S3. Antibodies list

Antibody	Type	Targeted species	Manufacturer	Cat. No.	Dilution ratio*
CFL2	Polyclonal	Rabbit	Lifespan Biosciences, Seatlle, WT, USA	LS-C409553	1:2,000
MyHC	Monoclonal	Mouse	DSHB, Iowa,IA, USA	MF20	1:1,000
MyoD	Monoclonal	Mouse	Santa Cruz Biotechnology, Dallas, TX, USA	sc-377460	1:1,000
MyoG	Monoclonal	Mouse	Santa Cruz Biotechnology, Dallas, TX, USA	sc-12732	1:1,000
YAP	Monoclonal	Rabbit	Cell Signaling Technology, Danvers, MA, USA	14074S	1:10,000
p-YAP	Polyclonal	Rabbit	Cell Signaling Technology, Danvers, MA, USA	4911S	1:10,000
Lamin B2	Monoclonal	Rabbit	Abcam, Cambridge, United Kingdom	ab151735	1:2,500
α-Tubulin	Monoclonal	Mouse	DSHB, Iowa,IA, USA	12G10	1:2,000
β-actin	Monoclonal	Rabbit	Sigma-Aldrich Chemical, St. Louis USA	A2066	1:10,000
Antibodies HRP-linked anti-rabbit IgG			Cell Signaling Technology, Danvers, MA, USA	#7074	1:10,000
Goat anti-mouse(H+L)			Invitrogen, Thermofisher Scientific, Waltham, MA USA	#32430	1:2,000

*All blots were visualized using a Femto reagent (Thermofisher Scientific).
